# Activation Associated ERK1/2 Signaling Impairments in CD8^+^ T Cells Co-Localize with Blunted Polyclonal and HIV-1 Specific Effector Functions in Early Untreated HIV-1 Infection

**DOI:** 10.1371/journal.pone.0077412

**Published:** 2013-10-15

**Authors:** Timothy Q. Crawford, Fredrick M. Hecht, Christopher D. Pilcher, Lishomwa C. Ndhlovu, Jason D. Barbour

**Affiliations:** 1 Hawaii Center for HIV/AIDS, John A. Burns School of Medicine, Department of Tropical Medicine, Medical Microbiology and Pharmacology, University of Hawaii at Manoa, Honolulu, Hawaii, United States of America; 2 HIV/AIDS Division, Department of Medicine, San Francisco General Hospital, University of California San Francisco, San Francisco, California, United States of America; Karolinska Institutet, Sweden

## Abstract

We recently observed that a large proportion of activated (CD38^+^HLA-DR^+^) CD8^+^ T cells from recently HIV-1-infected adults are refractory to phosphorylation of ERK1/2 kinases (p-ERK1/2-refractory). Given that the ERK1/2 pathway mediates intracellular signaling critical for multiple T cell functions, including key effector functions, the loss of ERK1/2 responsiveness may have broad consequences for CD8^+^ T cell function. In the current study, we hypothesized that the p-ERK1/2-refractory population, localized largely within the activated CD38^+^HLA-DR^+^ CD8^+^ T cell population, would display impairments in CD8^+^ T cell effector functions, such as cytokine production and degranulation, compared to CD8^+^ p-ERK1/2-responsive cells. We further hypothesized that the p-ERK1/2-refractory phenotype is persistent over time during untreated infection, and would correlate with poorer virologic control, in a manner independent of CD8^+^ T cell activation level. We performed single-cell resolution, flow cytometric assays of phospho-kinase responses paired to intracellular cytokine staining in one assay to examine IFN-γ, perforin and CD107α responses in CD8^+^ T cells by ERK1/2 signaling profile. On a per cell basis, p-ERK1/2-refractory cells, which fall predominantly within the activated CD8^+^ T cell compartment, produced less IFN-γ in response to polyclonal or HIV-1 antigen-specific stimulation, and expressed lower levels of perforin and CD107α. The p-ERK1/2 refractory cell population displayed minimal overlap with the PD-1 and Tim-3 inhibitory exhaustion markers and predicted high viral load independent of activation, suggesting that ERK1/2 may be a unique marker and point of intervention for improving CD8^+^ T cell function. Blunted effector functions, secondary to ERK1/2 signaling deficits concentrated within activated CD8^+^ T cells, may contribute to immunodeficiency and underlie the predictive capacity of CD8^+^ T cell activation on HIV-1 disease progression. (270/300).

## Introduction

CD8^+^ T cells are not directly infected during HIV-1 infection, but nonetheless exhibit profound functional deficits, alongside a highly skewed maturation profile, and accumulation of a population of highly activated CD8^+^ T cells [1]–[3]. Individuals who spontaneously contain virus replication in the absence of anti-retroviral treatment (ART), display low T cell activation levels [4]–[6] and exhibit maintenance of CD8^+^ T cell effector functions, including proliferative capacity, the ability to produce multiple cytokines (polyfunctionality), and elevated cytotoxic activity [7]–[9]. An expanding body of evidence points towards the quality of CD8^+^ T cell effector functions, including the ability to produce IFN-γ, express cytotoxic molecules such as perforin, granzymes and surface CD107α, as a key factor limiting viral replication [10]–[13]. Defects in these CD8^+^ T cell functions in HIV-1 disease contribute to the development of immunodeficiency.

HIV-1 disease is characterized by elevated, persistent immune inflammation, which drives a suite of changes to the immune system and solid tissues of the body [14]. Elevated expression of the ecto-NADase, CD38 and the class II human leukocyte antigen HLA-DR on the surface of circulating CD8^+^ T cells are commonly used as ‘activation’ markers tracking HIV-1-driven immune inflammation levels. High CD8^+^ T cell activation independently predicts rapid disease progression and poor disease outcome both in untreated HIV-1-infected adults and those on anti-retroviral therapy [15]–[18].

We recently observed that during early, untreated HIV-1 infection, the majority of activated (CD38^+^HLA-DR^+^) CD8^+^ T cells display a deficit in their ability to phosphorylate the extracellular signal-regulated kinases ERK1 and ERK2 (p-ERK1/2-refractory CD8^+^ T cells), while non-activated cells rarely displayed this signaling deficit [19]. In patients with higher levels of immune activation, a quarter or more of all CD8^+^ T cells display the ERK1/2 deficit, suggesting these cells may be impaired in their ability to respond to their cognate antigens. ERK1/2 proteins are critical mediators of intracellular signaling pathways, regulating multiple T cell functions such as proliferation, differentiation, and cytokine production [20]–[22]. Abrogation of ERK1/2 signaling in a large fraction of CD8^+^ T cells could have multiple deleterious functional consequences, including reduced T cell proliferation, altered differentiation profiles, changes to apoptotic programs, and altered effector functions [20], [22], [23].

In the current study, we hypothesized that p-ERK1/2-refractory CD8^+^ T cells would exhibit reduced effector function compared to p-ERK1/2-responsive cells. To test this hypothesis, we combined single-cell phospho-kinase flow cytometry [24], with intracellular cytokine staining [25], [26], to examine IFN-γ production, perforin content and CD107α in CD8^+^ T cells by ERK1/2 signaling profile. We examined differences in the percent of responding cells, and critically, the per cell expression levels of IFN-γ, perforin, and CD107α, as qualitative measurements of effector capacity. On a per cell basis, in recently HIV-1 infected adults, p-ERK1/2-refractory cells produced less IFN-γ in response to polyclonal or HIV-1 Gag stimulation, and exhibited lower cytotoxic capacity.

## Materials and Methods

### Clinical Cohort

We selected frozen PBMC specimens isolated from adults enrolled in the University of California, San Francisco OPTIONS project, a well characterized population of adults in known stages of HIV-1 infection. In order to examine early steps in the HIV immunopathogenic processes that drive later disease, we chose to examine anti-retroviral-naïve patients during a narrow window of early HIV-1 disease prior to severe immune decline. The majority of patients were between month 2 and 4 from the estimated date of HIV-1 infection [27], with a subset followed longitudinally up to 2.5 years before initiating treatment. Working in this narrow window of early infection, we observed HIV-1 driven disease processes in a comparatively intact immune system in the absence of clinical intervention.

### Ethics Statement

This study was based on de-identified, anonymized specimens that were collected as part of a larger longitudinal clinical cohort study. The parent study received approval from the University of California San Francisco’s Committee on Human Research institutional review board. All subjects gave written informed consent to participate in this study, and specifically authorized the use of their cells for in vitro studies of T cell function and activation.

### Cell Culture, Stimulation, Staining and Flow Cytometry Analysis

Cryopreserved PBMC were thawed into complete 10% complete RPMI media (RPMI-1640 supplemented with 10% FBS, 1% Penicillin/Streptomycin, 10 mM HEPES, 2 mM L-Glutamine (all Hyclone) and 10 µg/mL DNAse1 (Sigma). Cell concentrations normalized to 5E6 cells/mL and 1E6 viable cells (200 µL) were aliquoted per well into 2 wells of a 96-well U bottom plate (Corning). For the polyclonal stimulation assay, PBMC were rested for 14 hours at 37°C in a 5% CO2 incubator, then stimulated for 2 hours with 100 ng/mL PMA and 1 µg/mL ionomycin (PMA+I) with 5 µg/mL brefeldin A and 5 µg/mL monensin (BFA+M) (all Sigma). After 2 hours the cells were stained for viability with aqua amine reactive dye (AARD, Life Technologies) and extracellular surface markers: anti-CD3 Alexa700 (BD Biosciences (BD)) clone SP34-2), anti-CD4 PE-TexasRed (Life Technologies clone S3.5), anti-CD8 Pacific Blue (BD clone 3B5), anti-CD38 PE-Cy5 (BD clone HIT2), and anti-HLA-DR APC-H7 (BD clone L243) to identify activated T cell subsets. The PBMC were then re-stimulated 20 minutes with PMA+I, fixed in 4% paraformaldehyde (PFA, Electron Microscopy Sciences), permeabilized with custom phosflow buffer #643435 (BD), and stained for intracellular signaling and effector function markers: anti-phospo-ERK1/2 (pT202/pY204) Alexa488 (BD clone 20A), anti-IFN-γ PE-Cy7 (BD clone B27), anti-Perforin PE (AbCam clone D48). Alternatively, for HIV-1 Gag assay, cells were rested for 4 hours, stimulated 12 hours with 4 µg/mL/peptide HIV-1 Con B Gag Peptides- Compete Set (AIDS Research and Reference Reagent Program) with BFA+M, then processed identically as the polyclonal assay (AARD and surface-stained, stimulated for 20 minutes with PMA+I, fixed, permeabilized, and stained for intracellular markers). For measurements of PD1, Tim-3 and the longitudinal analysis, cells were rested for 16 hours without BFA+M, stimulated for 20 minutes with PMA+I, and then processed identically to the polyclonal and Gag assays, except for the staining panels, which employed anti-PD1 APC (BD clone MIH4) or anti-Tim-3 PE (R&D Systems clone 344826) as surface markers, and not stained for IFN-γ or perforin.

Fluorescence minus one (FMO) samples were prepared for each antibody conjugate on a single subject to facilitate gating. Single-stain compensation controls were prepared for each antibody-fluorochrome conjugate using anti-mouse beads (BD) or cells (anti-CD3 clone SP34-2 and AARD). Data was acquired on a custom 4-laser, 16-color BD Fortessa instrument. Compensation and analysis were performed in FlowJo (Treestar).

### Statistical Analysis

Statistical analysis was performed using GraphPad Prism statistical software (GraphPad Software, San Diego, CA) and SAS statistical software package (SAS). The non-parametric Mann-Whitney U was used for comparison tests and the Spearman Rank test was used for correlation analyses. Measures of central tendency are expressed as median and inter-quartile range (IQR 25^th^, 75^th^ percentile).

Visualization of longitudinal trends in the p-ERK1/2 refractory phenotype, and its association with viral load were displayed via a lowess fit of all patient data over time (Graphpad/Prism). Longitudinal, mixed effects statistical modeling was performed in the SAS System Proc Mixed procedure. The Proc Mixed procedure allows an assessment of the relationship of the p-ERK1/2-refractory phenotype to longitudinal viral loads and CD4^+^ T cell counts, and in adjusted models, and determination of whether the relationship of the p-ERK1/2-refractory phenotype with viral load and CD4+ T cell count is independent of CD8^+^ T cell activation levels.

## Results

### Subject Characteristics

Table 1 lists the clinical cohort characteristics for the 49 recently HIV-1 infected anti-retroviral treatment naïve individuals we studied. We evaluated 19 individuals for polyclonal responses, and a separate 30 individuals for HIV-1 Gag responses. PBMC for these experiments were taken from the first available clinical sample drawn at study entry. All patients were male, with a median age of 37 years, and had been infected with HIV-1 for an estimated median of 103 days. The subset of patients evaluated in this study did not differ significantly from the body of the UCSF Options cohort [19].

**Table 1:** Study Participant Characteristics.

Characteristic	Median [IQR†]
Age	37.0 [28.0, 43.5]
HIV-1 RNA (log_10_ copies/mL)	4.8 [4.1, 5.0]
CD4^+^T cell Count (cells/µL)	532 [406, 680]
CD8^+^, % CD38^+^HLA-DR^+^	24.8 [15.4,37.5]
Gender % Male	100
On ART†† at Time of Study (%)	0
Time Since Infection (Days)	103 [72,137]

† IQR = Interquartile Range.

†† ART = Antiretroviral Treatment.

### Blunted Effector Function by Activated p-ERK1/2-refractory Cells in Response to Polyclonal Stimulation

To examine CD8^+^ T cell effector function by ERK1/2 signaling ability, we used a flow cytometry assay developed in our laboratory that combines phospho-flow and ICS assays into a single experimental protocol (Crawford et al 2013, Submitted) (Materials and Methods). This approach allows quantification of both intracellular ERK1/2 phosphorylation and IFN-γ expression responses together within single cells. PBMC from 19 recently HIV-1-infected, treatment naïve adults, were stimulated with PMA+I for 140 minutes, and CD8^+^ T cells were examined for functional marker expression.

**Figure 1:**
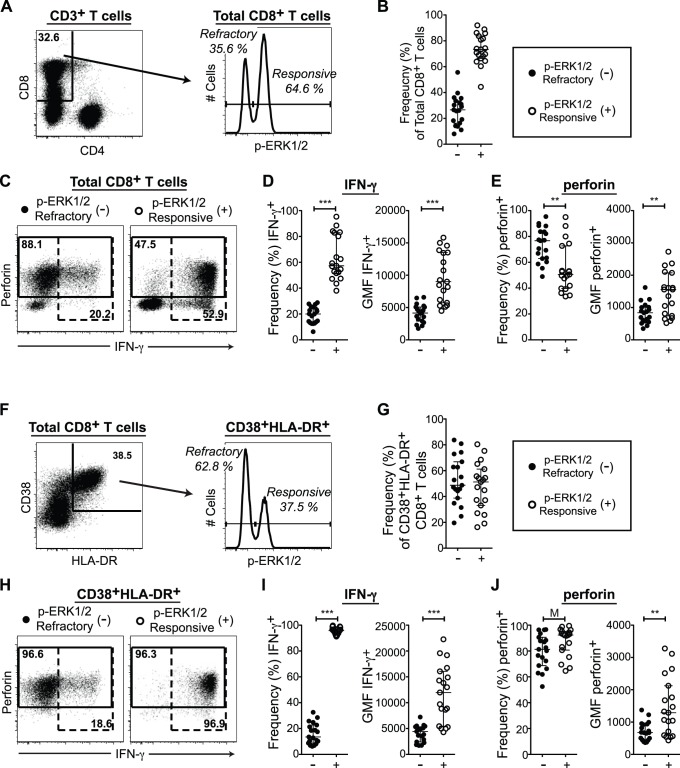
p-ERK1/2-Refractory CD8^+^ T cells exhibit low per cell effector function in response to polyclonal stimulation. (A–E) Response of total CD8+ T cells to140 minutes PMA+I. (**A**) Gating for CD8^+^ p-ERK1/2-refractory versus responsive T cell subsets. (B) Frequency of p-ERK1/2-refractory cells. (C) Gating for IFN-γ (dashed gates) and perforin (solid gates) expression within p-ERK1/2 subsets. (D) IFN-γ expression by ERK1/2 signaling response. Left graph displays frequency of IFN-γ^+^ cells contained within the parent population. Right graph, the IFN-γ geometric mean fluorescence intensity (GMF) of IFN-γ^+^ cells (E) Perforin expression by ERK1/2 signaling response. Left graph, frequency of perforin^+^ cells. Right graph, perforin GMF of perforin^+^ cells. (F–G) Response of highly activated (CD38+HLA-DR+) CD8+ T cells. (F) Gating for p-ERK1/2-refractory versus responsive subsets. (G) Frequency of p-ERK1/2-refractory cells within the CD38+HLA-DR+ compartment. (H) Gating for IFN-γ and perforin expression within activated p-ERK1/2 subsets. (I) IFN-γ expression by ERK1/2 signaling response: Left graph, frequency of IFN-γ^+^ cells. Right graph, IFN-γ GMF of IFN-γ^+^ cells. (J) Perforin expression by ERK1/2 signaling response. Left graph, frequency of perforin^+^ cells. Right graph, perforin GMF in perforin^+^ cells. Significance Not Significant (NS) p>0.01, Marginal (M) p<0.01, *p<0.05, **p<0.005, ***p<0.0005. (A,C,F,H, n = 1; D,E,I,J, n = 19).

The CD8^+^ T cell compartment exhibited a distinct bimodal ERK1/2 phosphorylation response following PMA+I stimulation (Figure 1A) [19]. A substantial population of cells in all patients remained refractory to PMA+I-induced ERK1/2 phosphorylation (p-ERK1/2-refractory cells) (Figure 1B, median p-ERK1/2-refractory frequency [IQR] = 26.6 [16.3,33.6]). Total CD8^+^ T cells were gated into p-ERK1/2-refractory and responsive populations based on their density along the p-ERK1/2 axis (gates in Figure 1A histogram). IFN-γ and perforin expression levels were then compared between these p-ERK1/2-refractory and responsive subsets (representative response and gates in Figure 1C). Expression was quantified first using frequency of parent as a comparison of the relative capacity of each subset to express IFN-γ or perforin, and second, the geometric mean fluorescence intensity (GMF) of IFN-γ^+^ or perforin^+^ populations was used to represent expression levels on a per cell basis.

Upon polyclonal stimulation, the capacity of p-ERK1/2-refractory cells to express IFN-γ was clearly and significantly impaired relative to p-ERK1/2-responsive cells. The p-ERK1/2-refractory compartment displayed a reduced frequency of IFN-γ^+^ cells, and those cells that did produce IFN-γ expressed significantly lower levels of IFN-γ on a per cell basis, compared to IFN-γ^+^ p-ERK1/2-responsive cells (Figure 1D and Table 2). The p-ERK1/2-refractory compartment exhibited a significantly greater frequency of perforin^+^ cells. However, perforin^+^ p-ERK1/2-refractory cells expressed less perforin per cell, than perforin^+^ p-ERK1/2-responsive cells (Figure 1E and Table 2).

**Table 2:** Polyclonal CD8^+^ T Cell Effector Capacity by ERK1/2 Signaling Ability.

Total CD8^+^ T Cells	p-ERK1/2-Refractory Median [IQR†]	p-ERK1/2-Responsive Median [IQR†]	Significance(p-value)
IFN-γ^+^(%)	20.0 [13.2,25.3]	57.3 [51.6,82.9]	<0.0001
IFN-γ^+^GMF ††	4146 [2914,5082]	9005 [5733,13520]	<0.0001
perforin^+^(%)	76.7 [63.1,83.9]	50.6 [39.8,72.7]	0.0012
perforin^+^GMF ††	839 [587,1011]	1558 [741,2073]	0.0061
**Within Highly Activated (CD38^+^HLA-DR^+^) Subset**			
IFN-γ^+^(%)	13.5 [8.7,22.6]	95.8 [93.8,97.1]	<0.0001
IFN-γ^+^GMF ††	4436 [2409,5335]	11969 [5443,16064]	<0.0001
perforin^+^(%)	81.3 [68.7,90.6]	92.3 [80.7,95.2]	0.0597
perforin^+^GMF ††	674 [449,938]	1290 [593,2124]	0.0035

† IQR = Interquartile Range.

†† GMF = Geometric Mean Fluorescence Intensity.

We then identified only the highly activated (HLA-DR^+^CD38^+^) CD8^+^ T cell population (gating in Figure 1F), and applied the same analysis as in total CD8^+^ T cells (Figure 1G–J). p-ERK1/2-refractory cells made up nearly half the activated CD8^+^ T cell population, (Figure 1G, median p-ERK1/2-refractory frequency [IQR] = 48.7 [38.8,66.9]), consistent with their localization to activated compartments [19]. Activated p-ERK1/2-refractory cells were extremely poor producers of IFN-γ, while nearly all activated p-ERK1/2-responsive cells expressed IFN-γ robustly (Figure 1I and Table 2). The majority of activated cells expressed perforin, and the p-ERK1/2-refractory subset exhibited only a marginally significant trend towards lower perforin^+^ frequency. However, perforin^+^ p-ERK1/2-refractory cells still expressed significantly less perforin on a per cell basis (Figure 1J and Table 2).

### Blunted Effector Function by p-ERK1/2-refractory Cells in Response to HIV-1 Gag

**Figure 2:**
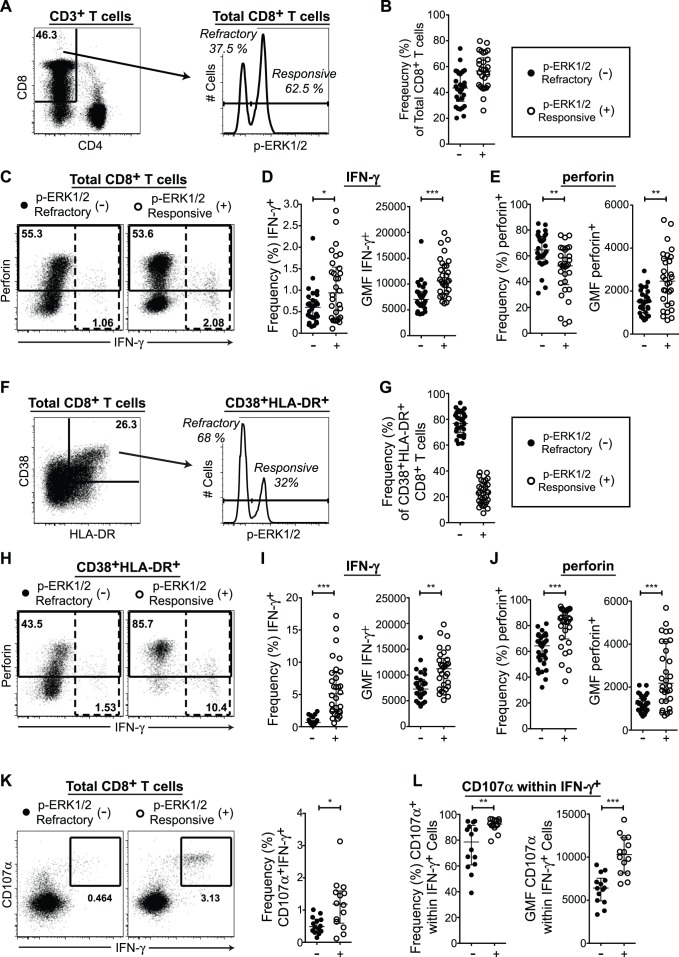
p-ERK1/2-refractory CD8^+^ T cells exhibit low per cell effector function in response to HIV-1 Gag stimulation. (A–E) Response of total CD8^+^ T cells to 12 hours HIV-1 Gag peptides and 20 minutes PMA+I. (**A**) Gating for CD8^+^ p-ERK1/2-refractory versus responsive T cell subsets. (B) Frequency of p-ERK1/2-refractory cells. (C) Gating for IFN-γ (dashed gate) and perforin expression (solid gate) within p-ERK1/2 subsets. (D) IFN-γ expression by ERK1/2 signaling response. Left graph displays frequency of IFN-γ^+^ cells contained within the parent population. Right graph, the IFN-γ geometric mean fluorescence intensity (GMF) of IFN-γ^+^ cells (E) Perforin expression by ERK1/2 signaling response. Left graph, frequency of perforin^+^ cells. Right graph, perforin GMF of perforin^+^ cells. (F–J) Response of highly activated (CD38^+^HLA-DR^+^) CD8^+^ T cells. (F) Gating for p-ERK1/2-refractory versus responsive subsets. (G) Frequency of p-ERK1/2-refractory cells within the CD38^+^HLA-DR^+^ compartment. (H) Gating for IFN-γ and perforin expression within activated p-ERK1/2 subsets. (I) IFN-γ expression by ERK1/2 signaling response: Left graph, frequency of IFN-γ^+^ cells. Right graph, IFN-γ GMF of IFN-γ^+^ cells. (J) Perforin expression by ERK1/2 signaling response. Left graph, frequency of perforin^+^ cells. Right graph, perforin GMF in perforin^+^ cells. (K–L) CD8^+^ T cells, (K) Gating for IFN-γ^+^CD107α^+^ expression and frequency of IFN-γ^+^CD107α^+^ cells within p-ERK1/2 subsets. (M) CD107α expression within IFN-γ^+^ cells by ERK1/2 signaling response: Left graph, frequency of CD107α^+^ cells. Right graph, CD107α GMF of CD107α^+^ cells. Significance Not Significant (NS) p>0.01, Marginal (M) p<0.01, *p<0.05, **p<0.005, ***p<0.0005. (A,C,F,H,K, n = 1; D,E,I,J, n = 30; L,M, n = 14).

We assessed CD8^+^ T cell responses to HIV-1 Gag peptide stimulation by ERK1/2 signaling ability in 30 recently HIV-1-infected, treatment-naïve adults that had not been previously studied, stimulating with HIV-1 p24 Gag peptides for 12 hours, then with PMA+I for 20 minutes to identify p-ERK1/2-refractory cells. We first compared the responses of total CD8^+^ T cells gated into refractory and responsive compartments (Figure 2A–E). Similar to the results obtained with polyclonal stimulation, we observed that the p-ERK1/2-refractory CD8^+^ T cell compartment contained significantly fewer IFN-γ^+^ cells than the p-ERK1/2-responsive compartment, and that IFN-γ^+^ p-ERK1/2-refractory cells expressed significantly less IFN-γ on a per cell basis (Figure 2D and Table 3). The frequency of perforin^+^ cells was again significantly higher in the p-ERK1/2-refractory compartment, while perforin expression levels in perforin^+^ cells per cell were significantly lower (Figure 2E and Table 3).

**Table 3:** HIV-1 Gag CD8^+^ T Cell Effector Capacity by ERK1/2 Signaling Ability.

Total CD8+ T Cells	p-ERK1/2-RefractoryMedian [IQR†]	p-ERK1/2-ResponsiveMedian [IQR†]	Significance(p-value)
IFN-γ^+^(%)	0.60 [0.37,0.79]	0.94 [0.35,1.54]	0.0421
IFN-γ^+^GMF††	7027 [5828,8512]	10585 [8210,13665]	<0.0001
perforin^+^(%)	64.6 [57.5,64.6]	52.3 [37.7, 65.0]	0.0006
perforin^+^GMF††	1513 [995,1873]	2467 [1412,3527]	0.0009
IFN-γ^+^CD107α^+^(%)	0.48 [0.33,0.72]	1.19 [0.59,1.59]	0.014
**Within IFN-γ^+^Subset**			
CD107α (%)	78.55 [60.8,91.4]	92.5 [90.7,95.7]	0.0033
CD107α GMF††	6409 [4924,7551]	10293 [8147,12252]	0.0002
**Within Highly Activated (CD38^+^HLA-DR^+^) Subset**			
IFN-γ^+^(%)	0.67 [0.14,0.96]	5.1 [2.36,8.4]	<0.0001
IFN-γ^+^GMF††	7278 [6144,9127]	11204 [7625,13235]	0.0005
perforin^+^(%)	64.3 [52.4,72.2]	84.6 [69.6,91.9]	<0.0001
perforin^+^GMF††	1213 [886,1475]	2183 [1350,4131]	<0.0001

† IQR = Interquartile Range.

†† GMF = Geometric Mean Fluorescence Intensity.

Within highly activated HLA-DR^+^CD38^+^ CD8^+^ T cells (gates in Figure 2F), p-ERK1/2-refractory cells represented over three quarters of the highly activated CD8^+^ T cell population (Figure 2G, median p-ERK1/2-refractory frequency [IQR] = 76.8 [70.0,84.4]. The significant difference in IFN-γ^+^ frequency between the p-ERK1/2-refractory and responsive compartments was even more profound in activated cells than in total CD8^+^ T cells, and lower levels of per cell IFN-γ expression was again evidenced in IFN-γ^+^ p-ERK1/2-refractory cells (Figure 2I and Table 3). The frequency of perforin^+^ cells was significantly lower in activated p-ERK1/2-refractory cells, and perforin^+^ p-ERK1/2-refractory cells expressed lower perforin per cell (Figure 2J and Table 3).

In 14 individuals with adequate specimen volume, we included anti-CD107α antibody during the stimulation with HIV-1 Gag peptides. CD107α is a membrane glycoprotein residing in cytotoxic granules that is exposed on the surface of the cell upon degranulation [28]. In total CD8^+^ T cells, the p-ERK1/2-refractory compartment exhibited significantly lower frequencies of double positive IFN-γ^+^CD107α^+^ cells (Figure 2K and Table 3). Within IFN-γ^+^ cells subset by p-ERK1/2-response, IFN-γ^+^ p-ERK1/2-refractory cells exposed less CD107α on their surface than IFN-γ^+^ p-ERK1/2-responsive cells, measured either as frequency or GMF (Figure 2L and Table 3).

### p-ERK1/2-refractory Cells are Distinct from Classical Exhaustion

**Figure 3:**
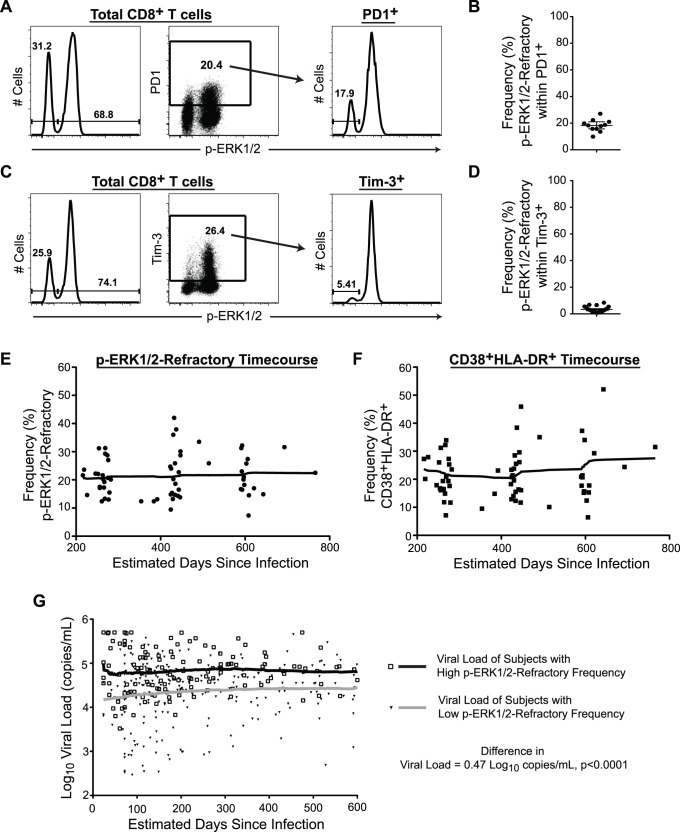
p-ERK1/2-refractory CD8^+^ T cells are distinct from classical exhaustion, remain stable over time and predict HIV-1 viral load. (A–D) CD8^+^ T cells following 20 minutes PMA+I**.** (A) Panels from left to right: ERK1/2 phosphorylation in total CD8^+^ T cells. Gating for PD1 expression. Gating for p-ERK1/2-refractory versus responsive subsets within the PD1^+^ compartment. (B) Frequency of p-ERK1/2-refractory cells within the PD1^+^ compartment. (C) Panels from left to right: The ERK1/2 phosphorylation response in total CD8^+^ T cells, Gating for Tim-3 expression in total CD8^+^ T cells. Gating for Tim-3 expression in total CD8^+^ T cells. (D) Frequency of p-ERK1/2-refractory cells contained within the Tim-3^+^ compartment. (E–F) Smoothed moving average plots displaying the frequency of p-ERK1/2-refractory (E) and CD38^+^HLADR^+^ (F) CD8^+^ T cells from HIV-1-infected treatment-naïve adults followed longitudinally over the first 2.5 years of infection. (G) Lowess plots displaying average viral load over time in patients stratified by high or low p-ERK1/2-refractory measurement at study entry. Open squares with black line represents individuals with a first clinical visit % p-ERK1/2-refractory CD8^+^ T cell measurement above the median frequency. Closed triangles with grey line represents individuals below the median frequency. Individuals with a high p-ER1/2-refractory measurement during early infection maintain significantly higher viral loads over time. (A,C n = 1; B n = 11; D, n = 20; E–F, n = 27 with 2–4 time points per individual, G, n = 74).

The increased expression of inhibitory receptors such as PD1 or Tim-3 on the surface of CTLs [29], [30] restrain T cell effector responses [31], and may contribute to a state of functional exhaustion [32], [33] in T cells during HIV-1 disease. To determine if p-ERK1/2-refractory state represented a new functional marker of ‘exhausted’ cells, we determined the extent of overlap between the p-ERK1/2-refractory compartment and PD1 or Tim-3 expression (Figure 3A–D). We observed that in all patients only a minor proportion of p-ERK1/2-refractory cells were positive for PD1 or Tim-3. The majority of cells exhibiting the ERK1/2 signaling deficit did not express these exhaustion markers.

### Stability of the p-ERK1/2 Refractory Population Over Time

In 27 individuals with available PBMC specimens from multiple time points drawn following study entry (200–800 days post-infection, all anti-retroviral-naive), we evaluated prevalence of the p-ERK1/2-refractory phenotype and activation state within the CD8^+^ T cell compartment over time. The frequency of p-ERK1/2-refractory CD8^+^ T cells remained stable and elevated over time (Figure 3C), while activation state exhibited a trend towards modest increase over time (Figure 3D).

### Strong Association of the p-ERK1/2-refractory Phenotype with Viral Loads Over Time

We then sought to assess the relationship between the p-ERK1/2 refractory phenotype as measured at a single point in early infection, for its ability to associate with longitudinal clinical markers in the absence of treatment. We included all subjects in the parent OPTIONS cohort for which we had ever obtained a standard phospho-flow measurement of the p-ERK1/2-refractory phenotype at the study entry time-point and also had longitudinal HIV-1 viral load and CD4^+^ T cell count data available for analysis. Seventy-four HIV-1 infected adults that fit these criteria (sixty from our previously published study identifying the ERK1/2 signaling deficit [19] and fourteen from the current study cohort not represented in our earlier report) had a p-ERK1/2 phenotype measurement, and longitudinal clinical marker data in the absence of treatment. The characteristics of this additional group of 74 subjects (97% male with a median age of 36 years, median HIV-1 viral load of 4.57 log_10_ copies/mL and median CD4^+^ T cell count of 533 cells/mL) did not differ significantly from the body of the OPTIONS cohort or the 49 individuals analyzed for effector activity in the current study (Table 1).

We performed longitudinal mixed effects models to assess the relationship of the pERK1/2-refractory phenotype to viral load. Briefly, we stratified the longitudinal data set by the median value of the p-ERK1/2-refractory phenotype (median 21.25% of CD8^+^ T cells), and CD8^+^ T cell activation (median 24.4% of CD8^+^ T cells), at the first time-point. We observed that patients with a p-ERK1/2-refractory phenotype value above the median had viral load levels of 0.47 log_10_ copies/mL higher (Figure 3E), and CD4^+^ T cell counts 53 cells/µL lower, than those with a lower p-ERK1/2 refractory phenotype. Likewise, we observed that patients with a CD8^+^ T cell activation value above the median had viral load levels of 0.39 log_10_ copies/mL higher, and CD4^+^ T cell counts 84 cells/µL lower, than those with a lower CD8^+^ T cell activation value at entry.

We then ran adjusted longitudinal mixed effects models to determine if the entry time-point p-ERK1/2 refractory phenotype would associate with longitudinal viral load and CD4^+^ T cell counts. We found that high p-ERK1/2-refractory values remained associated with higher viral loads (0.37 log_10_ copies/mL, p<0.0001), after adjustment for CD8^+^ T cell activation levels, which were also associated with higher viral load (0.27 log_10_ copies/ml, p<0.0001). We also observed that, after adjustment for CD8^+^ T cell activation levels, the p-ERK1/2-refractory level was no longer associated with lower CD4^+^ T cell counts over time (−25 cells/µL, p = 0.16), while higher CD8^+^ T cell activation remained associated (−76 cells/µL, p<0.0001) after adjustment for p-ERK1/2-refractory levels.

## Discussion

CD8^+^ p-ERK1/2-refractory T cells are highly abundant in recently HIV-1-infected treatment-naïve adults and are of increased in frequency in persons with high levels of CD8^+^ T cell activation [19]. In this study, we observed that p-ERK1/2-refractory CD8^+^ T cells exhibited a significantly reduced capacity to produce IFN-γ in response to polyclonal or HIV-1 Gag peptide stimulation relative to p-ERK1/2-responsive cells. The majority of p-ERK1/2-refractory cells expressed the cytolytic molecule perforin, but expressed lower levels of perforin per cell than p-ERK1/2-responsive cells. We also observed deficits in CD107α surface presentation, a specific marker of degranulation activity [28], within IFN-γ-producing CD8^+^ T cells from the p-ERK1/2-refractory compartment. Paired with the finding of poor IFN-γ and low perforin expression, it appears that p-ERK1/2-refractory CD8^+^ T cells have weakened cytokine and cytotoxic effector capacities on a per cell basis.

We found that persons with a higher fraction of p-ERK1/2-refractory CD8^+^ T cells during early infection displayed sustained higher levels of viremia, with up to one-half (0.5) log_10_ greater viral loads over time, an effect that was independent of CD8^+^ T cell activation levels. The p-ERK1/2-refractory phenotype was associated with a greater increase in viral loads than activation upon adjustment for activation level, but did not associate with CD4^+^ T cell counts after adjustment. However, higher CD8^+^ T cell activation levels remained associated with lower CD4^+^ T cell counts, suggesting the p-ERK1/2-refractory phenotype and immune activation may measure distinct components of HIV disease pathogenesis. Taken together, our findings suggest the p-ERK1/2-refractory phenotype may be an independent marker of T cell dysfunction in HIV disease, and may at least partially explain the link of CD8^+^ T cell activation levels to poorer clinical outcomes.

Perforin expression does not directly measure degranulation, but is required for cytotoxic killing and can be produced by HIV-1-specfic CD8^+^ T cells responding to antigen, representing marker of cytotoxic killing capacity [10], [34]. Low per cell perforin levels in p-ERK1/2-refractory CD8^+^ T cells may indicate a functional deficit in perforin expression during maturation, a failure to up-regulate perforin in response to antigen, or previous degranulation and release of perforin. Perforin expression within the p-ERK1/2-refractory compartment may reflect the differentiation/maturation state of p-ERK1/2-refractory cells, as perforin expression associated with memory-effector differentiation [13], [35]–[37]. We have previously observed their preferential localization within transitional memory compartments (CD45RA^−^CD28^−^CD27^+/−^) [19]. Together with the observation of low surface CD107α presentation by IFN-γ^+^ cells responding HIV-1 Gag, these data suggest reduced per cell cytotoxic potential of CD8^+^ T cells in the p-ERK1/2-refractory compartment.

Senescence or immune exhaustion of CD8^+^ T cells [33], [38], [39], shifts in maturation stage distributions [2], and expression of the inhibitory receptors PD-1 and Tim-3 [29], [30], are induced by chronic viral infection and compromise immune function. In this report we found that the p-ERK1/2-refractory phenotype did not significantly overlap with the PD-1 or Tim-3 expressing populations, as we had previously observed for Tim-3 [19]. We observed that the degree of difference in effector activity between p-ERK1/2-refractory and responsive cells tended to be somewhat enhanced in activated CD8^+^ T cells compared to bulk CD8^+^ T cells. The reasons for this are not clear, but may relate to additional functional features not captured by the p-ERK1/2-refractory phenotype, such as metabolic changes to CD38 (an NADase) expressing CD8^+^ T cells, maturation stage, or other features.

The ERK1/2 pathway has been shown to regulate IFN-γ production in T cells [40]–[42], through modulating activity of factors such as the activator protein-1 (AP-1) transcriptional complex [43]–[45]. AP1 binds transcriptional response elements within the *ifn-γ* promoter, stimulating *ifn-γ* transcription in response to not only HIV-1, but also other pathogens such as *Mycobacterium tuberculosis* (TB) [46], [47]. IFN-γ responses are a critical component of adaptive immunity controlling TB infection [48], [49], and TB is a major source of mortality among persons with HIV-1 infection [50]. However, the regulation of IFN-γ expression is more complex than a simple on/off switch mediated by one pathway (ERK1/2) or transcription factor complex (AP1). Differential epigenetic modifications (methylation and acetylation) of the *ifn-γ* promoter are associated with distinct T cell lineages [51] and differentiation states [52], [53]. These modifications alter chromatin structure around the *ifn-γ* promoter region and thus, transcription factor access to the promoter itself. Additionally, transcriptional regulation of *ifn-γ* is highly combinatorial, with described modulatory roles for CREB [46], T-Bet [54], NFAT [55], and STAT4 [56] in addition to ERK1/2. Further studies may evaluate changes in chromatin structure and transcriptional factor expression that may be complicit in the blunted ability of activated p-ERK1/2-refractory cells to produce IFN-γ during HIV-1 disease. Interventions to restore signaling through the ERK1/2 pathway may lead to expansion of effector capacities, distributed across a broad swath of T cell specificities.

Further study of the ERK1/2 signaling deficit is warranted, including assessment of responses to additional pathogens beyond HIV-1, and identification of the originating block or blocks leading to impairment of the ERK1/2 pathway. This block could be found at a specific upstream kinase, loss of a scaffold protein, induction of a phosphatase, or changes in expression of a surface receptor family needed for ERK1/2 pathway maintenance [57], [58]. Indeed, work from our group suggests that the impairment may extend to the ‘parallel’ MAPK pathway p38, and the impairment may occur at the level of MEK1/2 (data not shown), an upstream kinase, or at a point even further upstream. Finally, the block in the ERK1/2 pathway may not be a true impairment, but rather an aberrant over-expansion of a naturally occurring population of immature cells, or cells caught between differentiation states. HIV-1 is well known to induce a skewed T cell maturation and differentiation profile [59], [60]. Therefore, the functional deficit described here may be a consequence of changes to the signaling pathways mediating T cell development, such as the Wnt/β-Catenin pathway [61], [62], or selective defects in transcription factors, such as FOXO3A [63], [64], leading to altered or stagnant T cell maturation processes. In this latter case the appropriate therapeutic approach might not be direct targeting of the ERK1/2 pathway, but developmental maturation pathways such as Wnt/β-Catenin.

In summary, we found that a large subset activated CD8^+^ T cells, abundant in HIV-1 disease, have impairments in ERK1/2 signaling that colocalize with lower per cell effector function upon either polyclonal or HIV-1 Gag antigen-specific stimulation. This deficit in per cell function is independent of Tim-3 or PD-1 marker expression and affects up to a quarter or more of all CD8^+^ T cells in recently infected untreated HIV-1 positive persons. We observed that the ERK1/2 signaling deficit is stable over time during untreated infection, and persons with higher levels of p-ERK1/2-refractory CD8^+^ T cells have higher viral loads, independent of activation levels. The association of elevated CD8^+^ T cell activation with progressive HIV-1 infection may be directly linked to accumulation of large numbers of p-ERK1/2-refactory CD8^+^ T cells with poor per cell effector function, resulting in higher HIV-1 viral loads, and failure to resist or clear an array of infectious diseases other than HIV-1.
